# An Antioxidant Potential, Quantum-Chemical and Molecular Docking Study of the Major Chemical Constituents Present in the Leaves of *Curatella americana* Linn

**DOI:** 10.3390/ph11030072

**Published:** 2018-07-20

**Authors:** Mayara Amoras Teles Fujishima, Nayara dos Santos Raulino da Silva, Ryan da Silva Ramos, Elenilze Figueiredo Batista Ferreira, Kelton Luís Belém dos Santos, Carlos Henrique Tomich de Paula da Silva, Jocivania Oliveira da Silva, Joaquín Maria Campos Rosa, Cleydson Breno Rodrigues dos Santos

**Affiliations:** 1 Posgraduate Program of Pharmaceutical Innovation, Federal University of Amapá, 68902-280 Macapá, AP, Brazil; mayarafuji@hotmail.com (M.A.T.F.); elenilze@yahoo.com.br (E.F.B.F.); jocivania@unifap.br (J.O.d.S.); 2 Laboratory of Modeling and Computational Chemistry, Department of Biological and Health Sciences, Federal University of Amapá, 68902-280 Macapá, AP, Brazil; nsraulino@gmail.com (N.d.S.R.d.S.); ryanquimico@hotmail.com (R.d.S.R.); keltonbelem@hotmail.com (K.L.B.d.S.); 3 Laboratory of Toxicology, Department of Biological and Health Sciences, Federal University of Amapá, 68902-280 Macapá, AP, Brazil; 4 Computational Laboratory of Pharmaceutical Chemistry, Faculty of Pharmaceutical Sciences of Ribeirão Preto, 14040-903 São Paulo, Brazil; tomich@fcfrp.usp.br; 5 Department of Pharmaceutical Organic Chemistry, University of Granada, 18071 Granada, Spain; jmcampos@ugr.es

**Keywords:** *Curatella americana* L., natural antioxidant, quantum chemical, xanthine oxidase

## Abstract

Reactive oxygen species (ROS) are continuously generated in the normal biological systems, primarily by enzymes as xanthine oxidase (XO). The inappropriate scavenging or inhibition of ROS has been considered to be linked with aging, inflammatory disorders, and chronic diseases. Therefore, many plants and their products have been investigated as natural antioxidants for their potential use in preventive medicine. The leaves and bark extracts of *Curatella americana* Linn. were described in scientific research as anti-inflammatory, vasodilator, anti-ulcerogenic, and hypolipidemic effects. So, the aim of this study was to evaluate the antioxidant potentials of leaf hydroalcoholic extract from *C. americana* (HECA) through the scavenging DPPH assay and their main chemical constituents, evaluated by the following quantum chemical approaches (DFT B3LYP/6-31G**): Maps of Molecular Electrostatic Potential (MEP), Frontier Orbital’s (HOMO and LUMO) followed by multivariate analysis and molecular docking simulations with the xanthine oxidase enzyme. The hydroalcoholic extract showed significant antioxidant activity by free radical scavenging probably due to the great presence of flavonoids, which were grouped in the PCA and HCA analysis with the standard gallic acid. In the molecular docking study, the compounds studied presented the binding free energy (ΔG) values close each other, due to the similar interactions with amino acids residues at the activity site. The descriptors Gap and softness were important to characterize the molecules with antioxidant potential by capturing oxygen radicals.

## 1. Introduction

Free radicals or reactive oxygen species (ROS), including superoxide anions, hydroxyl radicals, and hydrogen peroxide are continuously generated in normal biological systems. Especially under stress, our body produces more ROS resulting in oxidative stress [1].

The intracellular production of ROS is associated with many cellular events including activation of enzymes such as xanthine oxidase (XO). This is a form of xanthine oxidoreductase, which is molybdenum-containing and uses O_2_ as the terminal electron acceptor during the purine metabolism; it is also implicated in human disease due to its capacity to generate uric acid and ROS such as hydrogen peroxide (H_2_O_2_) and superoxide (O_2_−) [2]. The XO has been involved in the pathogenesis of chronic heart failure, cardiomyopathy in diabetes and chronic wounds [3,4,5,6,7].

The inappropriate scavenging of these ROS or xanthine oxidase inhibition generally result in degradation of protein, lipid peroxidation, and DNA oxidation, which have been linked to aging, skin inflammatory disorders, many chronic diseases such as cancer, atherosclerosis besides contributing with the development of degenerative diseases as Alzheimer’s disease [1,8,9,10]. 

Several studies demonstrated that the intake of natural antioxidants has been associated with the promotion of health and prevention of diseases [11,12,13]. Natural antioxidants, including phenolic compounds, have diverse biological effects, such as anti-inflammatory, anti-carcinogenic and antiatherosclerotic effects, because of their antioxidant activity [14,15,16], many plants and their products have been investigated as natural antioxidants for their potential use in the preventive medicine.

*Curatella americana* L. is a member of the *Dileniaceae* family, popularly known in Brazil as “lixeira” or “cajueiro*-*bravo.” This species is an evergreen woody shrub that is characteristic of Neotropical Savanna, occurring from southern Mexico to Bolivia and in almost all savanna region of Brazil, [17,18,19]. Into Amazonian savanna *C. americana* it is one of the most frequent species found in Amapa, Amazonas, Para and Roraima states [20,21]. In the Brazilian folk medicine, it is used for inflammation, arthritis, bronchitis, high blood pressure [22]; the leaf decoction is used as an antiseptic and astringent [23]; the bark infusion is used for the treatment of cold, wounds healing and ulcers [24].

The extracts of the leaves and bark of *C. americana* are described in the literature for its anti-inflammatory, analgesic, antihypertensive, vasodilator, anti-ulcerogenic, antimicrobial, and hypolipidemic effects [25,26,27,28,29]. The computational investigation of natural compounds using molecular modeling by the DFT method allowed to evaluate chemical reactivity, molecular stability and molecular electrostatic potential maps (MEPs) to investigate the probable constituents with biological activity [30].

The aim of this study was to evaluate the antioxidant potentials of the hydroalcoholic extract of leaves of *C. americana* (HECA) through the scavenging DPPH assay, followed by the evaluation of the major chemical constituents by quantum chemical studies (DFT B3LYP/6-31G**), such as Maps of Molecular Electrostatic Potential (MEP), Frontier Orbital’s (HOMO and LUMO), multivariate analysis (PCA and HCA) and transference of electron compared with gallic acid to identify chemical descriptors that may characterize molecules with antioxidant potential. The molecular docking simulations with the xanthine oxidase enzyme (PDB codes 3 NRZ) was used to evaluate another possible action mechanism of the antioxidant potential in preventing ROS formation and relate the most promising molecules to their chemical characteristics.

## 2. Results and Discussion

### 2.1. Chemical Constituents and Molecular Modeling of Curatella americana L. 

**Figure 1:**
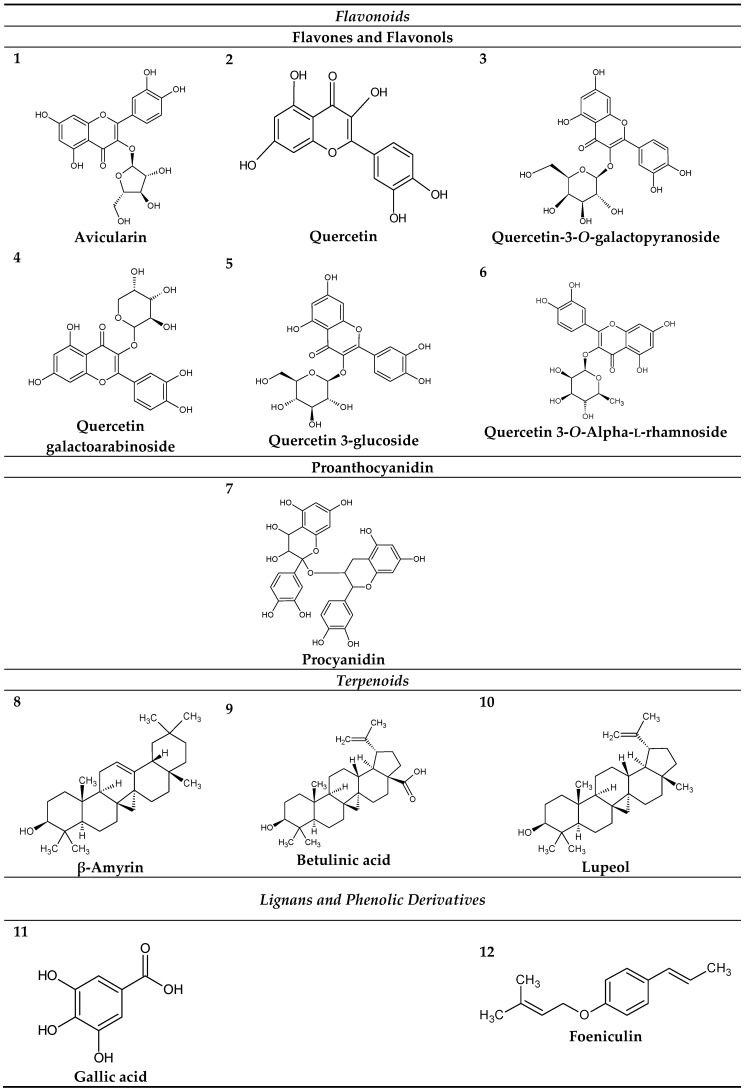
Chemical constituents of the leaves the *C. americana* L*.*

Phytochemical analyses of the leaves of *C. americana* L. already published, revealed the presence of phenolic compounds, flavonoids, terpenes, saponins and steroids according to studies performed by El-Azizi et al. and Gurni and Kubitzki [31,32], that identified the following compounds: (**1**) Avicularin; (**2**) Quercetin; (**3**) Quercetin-3-*O*-galactopyranoside; (**4**) Quercetin galactoarabinoside; (**5**) Quercetin-3-glucoside; (**6**) Quercetin-3-*O*-Alpha-l-rhamnoside; (**7**) Procyanidin; (**8**) β-Amyrin; (**9**) Betulinic acid; (**10**) Lupeol; (**11**) gallic acid; (**12**) Foeniculin (see Figure 1). The identified molecules were used in the molecular modeling study.

### 2.2. DPPH Scavenging Assay

**Figure 2:**
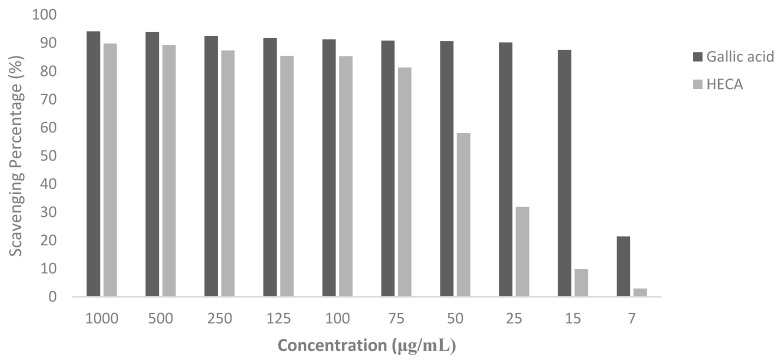
DPPH radical scavenging potential of gallic acid as the reference compound and hydroalcoholic extract of *Curatella americana* (HECA). The mean of scavenging percentage was significative different in all concentrations at *p* < 0.01.

The hydroalcoholic extract of the leaves of *C. americana* (HECA) is rich in phenolic compounds, mainly flavonoids, which have been proposed to exert beneficial effects in multiple disease states due to their antioxidant properties [11,13]. Flavonoids can scavenge a wide range of reactive oxygen species (ROS) by their classical hydrogen-donating antioxidant activity, as well as inhibition of ROS formation [33,34]. The DPPH scavenging potential of HECA and standard reference compound (positive control), Gallic acid, is presented in Figure 2.

With an estimative based in an exponential model, the 50% concentration inhibitory (IC_50_) was calculated, R^2^ = 0.8. Although there are a lot of antioxidant constituents in HECA, even in gallic acid, the activity was moderate, showing an IC_50_ of 45 μg·mL^−1^, lower than that previously identified by Lopes et al. [29], the differences in the methodology to obtain the hydroalcoholic extract must be considered. Besides that, it is important to emphasize that the control is an isolated substance and, therefore, would be indispensable to perform the quantum chemical studies to evaluate the most promising molecules with the antioxidant potential of HECA. Most of the compounds identified in the HECA were flavonoids admittedly as potent antioxidants, such as gallic acid (compound **11**) and quercetin (compound **2**); this fact can explain the great potential pharmacological of HECA mainly for the important hypolipidemic and anti-inflammatory activity of this species.

### 2.3. Molecular Modelling

#### 2.3.1. Maps of Molecular Electrostatic Potential (MEP) and Frontier Orbitals (HOMO and LUMO)

The maps of molecular electrostatic potential (MEP) show the regions featured by colors that vary depending on the potential/charges. The positive regions (blue/green) indicate the portion of the molecule with the higher probability to suffer a nucleophilic attack, while the negative regions (green/red) indicate the regions that will perform nucleophilic attacks [35]; see Figure S1 in the supplementary material.

The analyses of the *C. americana* L. constituents showed that the positive regions presented a uniformity or pattern, mainly due to the hydrogens (H) directly bonded to the oxygens atom (hydroxyls –OH). The positive electrostatic potentials maximum of the compounds varied from 0.02926 a.u (compound **12**) to 0.08821 a.u. (compound **2**). The compounds **8**, **9**, **10** and **12** presented the less positive regions, evidencing that in this portion there is a higher probability of occurring nucleophilic attacks than the other molecules.

There is a pattern in the distribution of the negative regions in the molecules, being located mainly in the hydroxyls (–OH) and oxygen atoms (O) bonded to other elements is explained by the higher oxygen electronegativity. Therefore, the minimum electrostatic potential for the molecules varied from −0.09952 a.u (compound **1**) to −0.04836 a.u. (compound **12**), confirming the qualitative analysis of the regions, wherein compound **1** presented less electrostatic potential (negative region); probably this region will perform more nucleophilic attacks than compound **12**. In addition, the variation of electrostatic potentials, the **2**–**6** and **7**–**12** compounds deserve some feature in the qualitative analysis by showing close values to compounds **1** and **11** (gallic acid), respectively.

The frontier orbitals are capable of providing information about the Highest Occupied Molecular Orbital (HOMO) energies and the Lowest Unoccupied Molecular Orbital (LUMO) energies, besides acting as electron-donors and electron-acceptors, respectively [36]. Thus, the higher the HOMO energy, the higher the capacity to realize nucleophilic attacks, that is, donating electrons; it is an important electronic parameter for describing the antioxidant ability because it can be related to electron transfer reactions [37,38].

Gallic acid (compound **11**) has demonstrated significant antioxidant effects and has been used as the standard in antioxidant assays [39]. Thereby, it is important to provide quantum chemical information about this compound to improve the search of others with similar antioxidant activity. The HOMO value for gallic acid was −1.2327 eV and the LUMO energy was 0.2414 eV; both were lower than others compounds values. In the HOMO, the regions are distributed in the entire molecule, while in the LUMO, these regions are concentrates in its aromatic ring (Figure S2). The analysis of the orbital HOMO showed that the regions are located predominantly over the aromatics rings (over the double bond) and isolated over the carbon atoms of this aromatic ring. Compound **9** presented the lowest value of HOMO = −6.4722 eV while **7** showed the highest value of HOMO = −0.0435 eV followed by the compounds **2** and **12**. It is important to emphasize that compounds **2** (Quercetin) is one of the most studied and potent antioxidant flavonoids that can scavenge ROS (metal- and nonmetal-induced), probably due to its free 3–OH, which to increase the stability of the radical flavonoid [40]. Therefore, compound **9** presents the lowest probability to perform nucleophilic attacks regarding the other compounds studied. Besides, compounds **8** (HOMO = −6.0213 eV) and **10** (HOMO = −6.3092 eV) presented values close to compound **9**, which may have the same capacity, confirming the qualitative analyses in MEP. 

Regarding the orbital LUMO analyses, they show the regions more susceptible to suffer nucleophilic attacks because of it character electron-acceptor, thus, the lower the energy of LUMO, the lower the resistance to accept electrons [41]. Even the LUMO does not show similarities or patterns when compared; it was possible to observe that the regions are close to the aromatic rings, over the hydrogen atoms (H) that stabilize the valence of carbons atoms (C) in these rings and mainly over the carbons that perform double bond in the aromatic rings. 

The minimum values (−0.1570 eV, 0.0065 eV) and maximum (0.7959 eV) were represented by compounds **12**, **2** and **8**, respectively. Thus, between the orbitals LUMO, compounds **8** and **10** (LUMO = 0.7619 eV) have the higher resistance to suffer nucleophilic attacks or accept electrons than the other ones.

#### 2.3.2. Multivariate Analysis PCA and HCA

After the determination of all molecular descriptors, the quantum chemical and QSAR variable values were auto-scored or standardized to give each variable an equal weight in mathematical terms to develop a multivariate analysis step. PCA was used to reduce the number of variables and select the most relevant ones, i.e., those responsible for classification of the compounds into two groups (more reactive and less reactive) based on the quantum chemical analysis of gap properties of the gallic acid, because the gap value indicates the chemical reactivity and molecular stability [42]. Good separation was obtained using five variables, see Table 1.

The model was constructed with the three principal components (PC1, PC2, and PC3) which describes 96.2259% of the overall variance as follows: PC1 = 74.6975%, PC2 = 15.1881% and PC3 = 6.3403%, losing just 3.7741% of the original information. PC1 contains 74.6975% of the original data, and the combination of the first two components (PC1 + PC2) contains 89.8856% of the total information. The principal components can be written as a linear combination of the selected descriptors. The mathematical expression for PC1 and PC2 are shown below:

PC1 = 0.4901HE + 0.5064LogP − 0.4281DMT + 0.4719GAP − 0.3121 (1/η),(1)

PC2 = 0.0978HE + 0.1308LogP − 0.4199DMT − 0.0329GAP + 0.8922 (1/η),(2)

The multivariate analysis (PCA and HCA) permitted to distinguish the compounds as the most reactive and less reactive through the stability measure, the GAP. This descriptor results from the difference between the LUMO-HOMO, compounds with low GAP values are generally reactive while molecules with high GAP values have higher molecular stability and lower reactivity [43]. A large gap implies good thermodynamic stability of the compound, whereas a small gap suggests an easy electronic transition, so we hypothesize is that the molecules with antioxidant potential are less stable and, therefore, more reactive.

**Figure 3:**
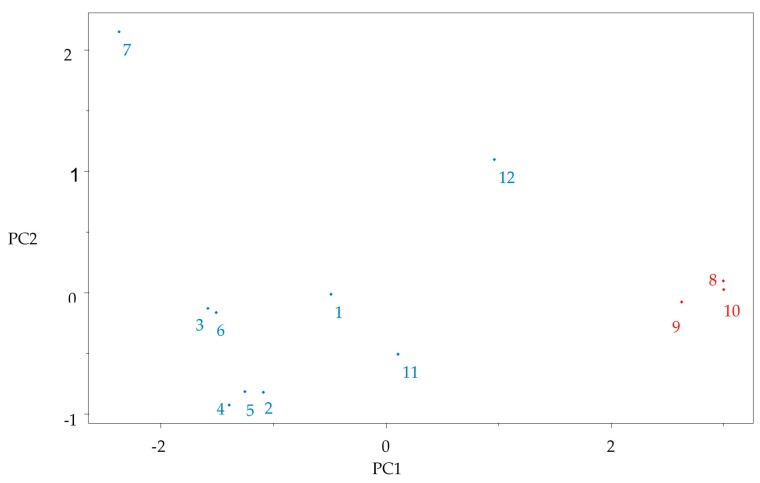
The plot of PC1–PC2 scores for *Curatella americana* chemical constituents. Red color indicates less reactive compounds and blue color indicate more reactive compounds.

**Figure 4:**
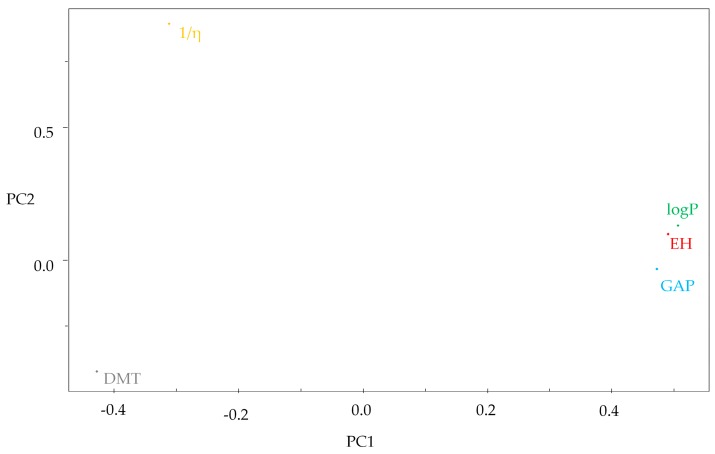
The plot of the PC1–PC2 loadings with the five descriptors selected.

The compounds with lowest GAP values varied between 1.47 eV (compound **11**—gallic acid) to 0.06 eV (compound **7**—procyanidin), see Table 1. These constituents are less stable and more reactive being grouped in the left (Figure 3). These compounds had higher contributions for the descriptors 1/η and DMT (Figure 4).

As a matter of fact, these compounds are flavones, flavonols and phenolic derivatives that through their ringed structures, conjugated double bonds and the presence of functional groups in the ring, can prevent the formation and scavenging of reactive oxygens species [40].

The phenolic compounds have been considered promising natural photoprotectors due to their potent antioxidant activity. Studies with cells and extracts of plants rich in polyphenols, including gallic acid, procyanidin and a mixture of flavonoids, demonstrated the protective action against the UV-induced damage in DNA through direct antioxidant action [44,45].

The values show that compounds **8**, **9** and **10** are the lowest reactive in the leaves of *Curatella americana*, due to the highest values of GAP (6.81 eV, 6.64 eV, and 7.07 eV, respectively), being grouped in the PCA at the right portion of the graph (Figure 3). These compounds had contributions from the descriptors LogP and HE; it is interesting to note that they had the lowest capacity to donate electrons due to their HOMO. 

The hydration energy (HE) is related to the drug capacity to absorb or release energy when in contact with an aqueous solvent. It is important to infer the transport and distribution for the different plasmatic biophases, as well as attraction and interaction of the drug with its receptor. Negative values indicate an exothermic reaction while positive values indicate an endothermic reaction; in other words, the more negative the values for HE, the more water soluble the molecule. The HE negatives values of the compounds vary from −49.340 kcal/mol (compound **7**) to −0.640 kcal/mol (compound **10**), thus, the procyanidin (compound **7**), showed the best solubility. Besides, this compound showed a higher softness (1/η) value (30.8818 eV) and this descriptor was important for the grouping in the PCA. The molecular softness represents the facility a molecule to deform [40]. Therefore, the higher the softness, the lower the energy necessary for the transition electron from HOMO to LUMO probably generating more stable radicals after the donation of electrons in function of the possibility of the unpaired electrons be able to transit to the LUMO region with less energy.

Procyanidin has been considered an important antioxidant, presenting important effects in protecting human neutrophil and erythrocytes hemolysis caused by ROS [46,47]. In a double-blinded with two treatments, placebo and a control treatment study, the consumption of capsules rich in this compound for four weeks reduced bloody ambulatory pressure probably due to its antioxidant effects [48]. 

The lipophilicity coefficient (LogP) is a property that quantitatively measures the lipophilia of the compounds, one of the most important molecular properties for drug absorption [49]. It is possible to observe that there is a relation between HE and LogP, because of negative values of HE being related to the compounds’ hydrophilicity, while the positive values are related to lipophilicity and this characteristic is exposed by the LogP, i.e., when higher is the HE values, higher will be the LogP value. 

Therefore, the terpenoid compounds **8** (β-Amyrin) showed higher values for HE (0.250 kcal/mol) and consequently higher values for LogP (8.090) and hence, it is the most lipophilic molecule. The other two terpenoids, compounds **9**, **10** and compound **12** (foeniculum), although presenting negative values for HE, close to zero, showed positives values for LogP, having important lipophilicity too, see Table 1. These characteristics of the terpenoids added to the low HOMO values, small softness and the high GAP value, showing less reactivity and consequently higher stability, suggest that this class does not show important antioxidant activity by scavenging ROS, although studies performed with plants rich in these compounds have demonstrated a potent antioxidant activity [16], maybe the other compounds in the studied extracts contribute to this activity.

**Figure 5:**
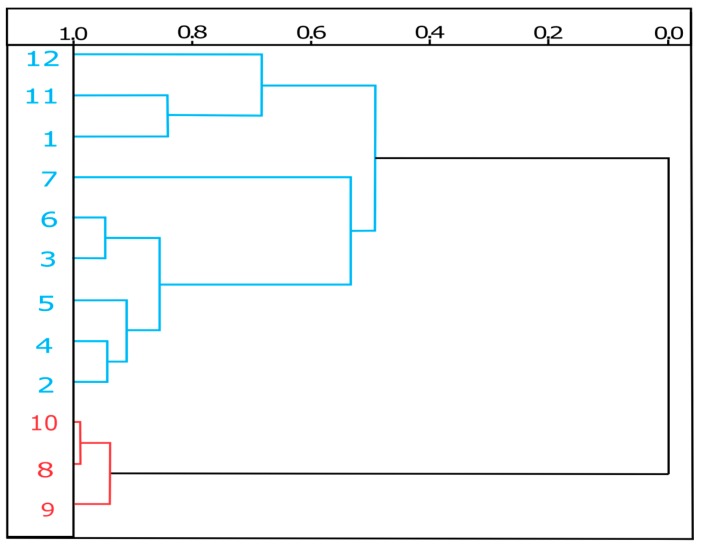
HCA dendrogram for *Curatella americana* constituents showing them separated into two main classes (red color indicates less reactive and blue color indicate more reactive compounds).

In the HCA technique, the distances between a pair of samples are computed and compared. Small distances imply that the compounds are similar, while non-similar samples will be separated by relatively large distances. The scale of similarity ranges from 0 for samples with no similarity to 1 for samples with great similarity. The dendrogram in Figure 5 shows that the HCA results confirmed the PCA analysis. 

#### 2.3.3. Theoretical Mechanism to Antioxidant Activity via Electron Abstraction

Antioxidant activity is highly related to the electron donation capacity. The hydrogen transfer step has been highlighted, but the antioxidant activity does not only depend on the energy strength of the O–H bond. The stabilization of the cation-radical and radical species formed should also be considered [50,51,52]. Therefore, we evaluated via ionization potential value (IP) and spin density compounds **7** (procyanidin), **11** (gallic acid) and **12** (foeniculin). Glycosylated flavonoids were disregarded because they had lower antioxidant activity than their free aglycones [53,54] and terpenoids because they had lower HOMO values and in the PCA analysis were grouped separately from the known antioxidant compounds. Procyanidin and foeniculin presented high HOMO values besides low Gap values, showing low molecular stability and high chemical reactivity.

The ionization potential (IP) represents the facility of an electron donation, and abstraction of electron; the molecules with the lowest IP values are more active. The ionization energies for a radical can be also used as a measure of stability of the corresponding cation, when the ionization energy is lower, the radical should be more stable [55,56]. The calculated values for the selected molecules are shown in Table 2.

According to the calculated IP values, molecule **7** (procyanidin) shows higher antioxidant activity than molecules **12** (foeniculin) and **11** (gallic acid), confirming the qualitative analyses of the multivariate analyses (PCA and HCA). Besides that, its radical is probably more stable.

**Figure 6:**
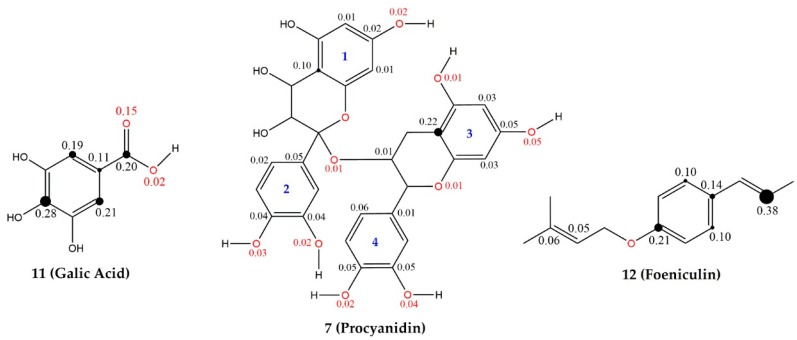
Spin densities in the cation free-radical of selected compounds.

An efficient antioxidant is one that donates the electron with ease and then in this process becomes more stable. The stabilization studied here is in function of the resonance process, after the donation, the molecule rearranges the electrons so that they do not to remain in the reactive radical form. The density spin shows the contribution of the atoms in the stabilization of the molecule, since we will have unpaired electrons. The resonance structures of cation free-radicals by electron abstraction can be observed by spin density distributions for the compounds selected, see Figure 6.

Gallic acid (**11**) presented higher spin distribution, ranging from 0.02 to 0.28 in the structure with global contribution in the benzene ring of 0.79, despite the presence of hydroxyls bound in the aromatic ring, they do not participate in the stabilization, only acetyl bound to the aromatic ring. However, gallic acid has the highest IP value (175.62 Kcal/mol) among the studied compounds.

Procyanidin had the lowest IP value (152.67 Kcal/mol) and, therefore, it was the molecule with greater ease for electron donation. Its radical cation presented a more uniform distribution of spin in the structure, with global contribution of the rings 1, 2, 3 and 4 from 0.14, 0.15, 0.33 and 0.17, respectively. Despite the low contribution of ring 2, it is possible to observe the stabilization and greater probability of electron output in this ring by HOMO (supplementary material Figure S2). It is important to note that the cation radical formed from this molecule presents lower energy indicating greater stability, it is possible that the more uniform distribution of the electrons in the molecule after the exit of one electron contributes to radical stability, this behavior can be related to the high softness of this molecule that could allow better electronic transition.

In relation to the structure of Foeniculin (**12**), there was a greater contribution of propenyl among the analyzed structures (value of 0.38). The resonance occurs with the hyperconjugation of the carbon of the radical, thus sacrificing an electron for the donation, after the presence the aromatic ring leaves the molecule stable due to resonance.

#### 2.3.4. Molecular Docking Study

To evaluate the action mechanism of potential antioxidants in preventing ROS formation, we performed a molecular docking study of the compounds with the xanthine oxidase enzyme. According to the studies of Cao et al. [2], hypoxanthine is first hydroxylated by XO at C-2 to form xanthine and then converted it to uric acid, in a reductive half-reaction in which the substrate is oxidatively hydroxylated at the molybdenum (Mo) center, hence, also generating ROS. The inhibition of xanthine oxidase would be a strategy to treat and prevent diseases that result in the accumulation of uric acid and consequently the ROS [57,58].

Although the most known inhibitor drug of XO has been allopurinol, we chose using febuxostat as the reference, because the allopurinol behaves as an analog of hypoxanthine, which is self-oxidation to form oxypurinol (the active inhibitory metabolite) resulting in the reduction of O_2_−, so remaining the production of ROS [58]. Instead, febuxostat is reported to be significantly more potent, probably because it fills the pocket of XO obstructing substrate binding. Thus, febuxostat should not be affected by enzyme redox state and interaction with XO and does not induce ROS formation [59].

**Figure 7:**
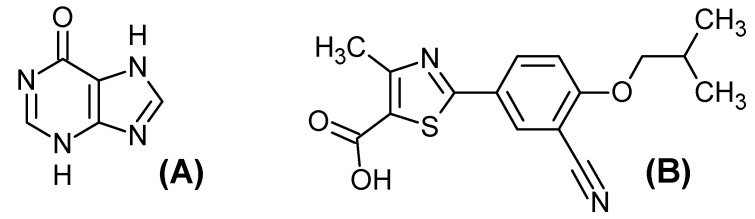
Control Ligands 2D structure: (**A**) hypoxanthine (HPX) and (**B**) Febuxostat (FBX).

The structure derived from X-ray of hypoxanthine complex with xanthine oxidase PDB codes 3 NRZ (*Bos taurus*) was selected for molecular docking studies of the HECA chemical constituents due to good parameters for experimental resolution (1.8 Å). The control ligands used in the molecular docking study were Hypoxanthine (HPX) and Febuxostat (FBX), Figure 7, downloaded at PDB server in sdf format ensuring the bioactive conformation. For validation of the docking method, the hypoxanthine structure with crystallographic information was submitted to docking until the best-docked ligand conformation that had a root mean square deviation (RMSD) of 1.64 Å. According to Hevener et al. [60], Santos et al. [61] and Cruz et al. [62] the binding prediction mode using the docking, affirm that when the RMSD is less than 2.0 Å on the crystallographic pose of the ligand can be considered satisfactory. Therefore, our results with the methodological proposal using these parameters are optimal and satisfactory.

The molecular docking method identified a conformation that allows the ligand also to interact with the active sites for hypoxanthine (PDB 3 NRZ) that around the α-helix between the amino acids residues 878–882, 1012–1014 and for β-sheet between the amino acids residues 801–805, 912–915, 1007–1011, 1076–1079. For the ligand, it is possible to see common hydrogen bonds with residues Arg880 and Glu802. There is also a hydrophobic interaction with residues Phe914, Phe1009, Ala1078 and Ala1079 as observed by Cao et al. [2].

**Figure 8:**
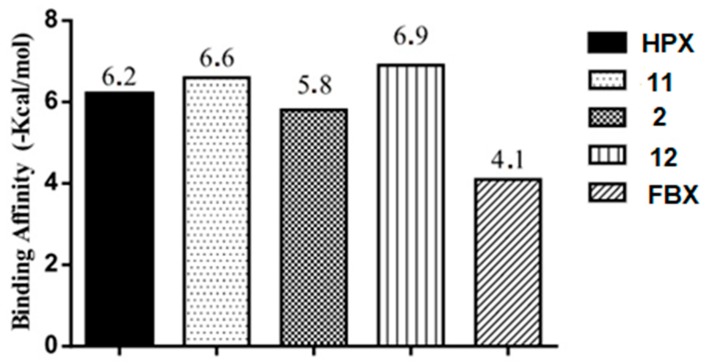
Binding affinity provided by AutoDock/Vina software of the compounds **11** (Gallic acid), **2** (Quercetin) and **12** (Foeniculin) and control ligand febuxostat (FBX). Ligand complexed hypoxanthine (HPX) for XO (organism *Bos taurus*).

The evaluation of affinity showed that foeniculin (compound 12) has a higher binding affinity (−6.9 Kcal/mol) in relation to the studied compounds and that it had a variation ±0.7 Kcal/mol in comparison to the HPX, and the other structure with better affinity, gallic acid (compound 11), had a variation of ±0.4 Kcal/mol, see Figure 8. 

However, gallic acid interacts with Glu1261 in the active site which is responsible for the deprotonation of the prosthetic group Mo-OH in the enzyme; this residue is universally conserved in the families of the enzymes containing molybdenum. After deprotonation, the nucleophilic attack occurs on the substrate promoting the oxidation reaction, generating ROS. In this way, gallic acid could behave as a competitive inhibitor of XO. Although foeniculin has high affinity, the presence of the highly reactive chemical group (the isoprene) and delocalization of π-type electrons (resonance), which would be rapidly oxidized by the enzyme generating ROS. The low gap (0.185 eV) of this molecule and the lowest positive electrostatic potentials, confirms its high chemical reactivity and it smaller capacity to suffer a nucleophilic attack.

**Figure 9:**
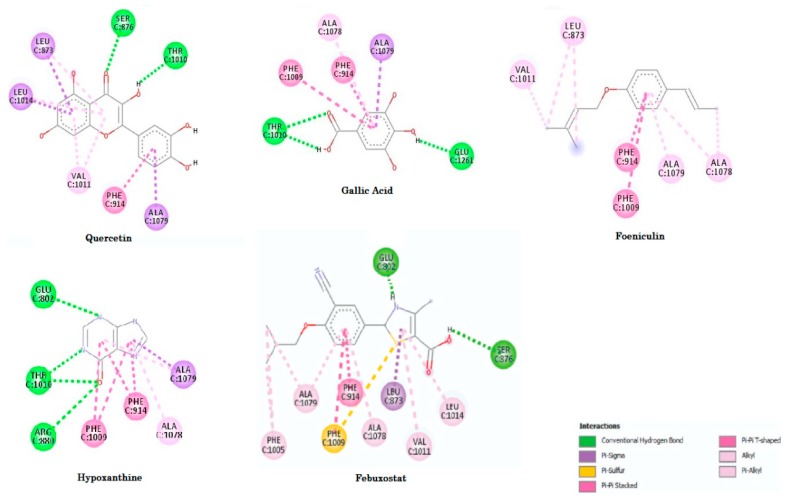
Interactions of the compounds with XO enzyme. Nominal interactions, aminoacids, and distances can be seen in Table 3.

We can also observe higher interaction numbers of febuxostat, which is a potent inhibitor of the enzyme xanthine oxidase. We found similar interactions with those published by Okamoto et al. [59], except for the interaction with the Ser876, Ala1078, and Ala1079. The interaction Ala1079 was found in all compounds selected, but the Ser876 only was found in the febuxostat and quercetin (compound **2**). Figure 9 shows the interactions for the docked structures that had the best affinities. Amino acids residues, quantitative data of distances and binding free energies (ΔG) between the compounds and XO receptor are shown in Table 3.

By analyzing the interaction sites for quercetin (compound **2**, see Figure 9) and comparing with the interaction sites of the febuxostat, we observed that the results were similar with the active sites (XO) having amino acid residues which are around the α-helix in the Ser876, Leu1014; and for β-sheet in the amino acids residues Leu873, Phe914, Val1011 and Ala1079, that with exception of Thr1010 presented just with the quercetin (compound **2**) agree with the literature [2]. In fact, the quercetin has been demonstrated to be an important inhibitor of XO in vitro, probably behaving as the febuxostat preventing the access of the substrate in the active site of the enzyme [63,64].

It is possible to verify that, among the standard and control compounds (ligands HPX and FBX), the increase in the number of interactions would result in the lowering of binding free energy, which indicates a higher degree of the spontaneity of the interactions [65]. On the other hand, we observed that the FBX interacted with Leu873, Ser876, Val1011 and Leu1014 as the quercetin (compound **2**) which had the binding free energy lower than FBX while the foeniculin (compound **12**) that had a lowest binding free energy (ΔG = −7.13 kcal/mol), showed only Leu873 and Val1011 residues in common with FBX and quercetin, suggesting that these residues are the most important than the amount of interactions. It is important to emphasize that these four residues are in the solvent-accessible channel leading to the molybdenum center, explaining the potential inhibitor of quercetin [66].

It is interesting to note that the compounds do not have significant structural similarity; however, the compounds studied had binding free energy values approximate to each other, due to the similarity of the interactions with the amino acids residues.

## 3. Materials and Methods 

### 3.1. Plant Material

*Curatella americana* leaves were collected from Macapa in Amapa State, Brazil, in the month of february 2015. A sampling location (00°9′75.251′ S, 51′8′57.6733′′ W) was marked by a global position measuring (GPS Garmin nüvi 40). The scientific identification of the vegetable material was held in the Herbarium of the Federal University of Amapá under the registration number 010266 for future reference.

#### Preparation of Plant Extract

Dry and pulverized leaves were extracted with 70% ethanol, in the proportion of 1:3 (*w*/*v*). The crude extract was filtered in vacuum using Whattman^®^ filter. The hydroalcoholic extract was evaporated under vacuum rotatory evaporator (IKA^®^ RV 05 basic), lyophilized and kept at −20 °C in a freezer until further use.

### 3.2. DPPH Scavenging Assay

The 1,1-diphenyl-2-picrylhydrazine (DPPH) is a stable free radical that react with compounds that can donate a hydrogen atom that decolorizes the DPPH solution. Therefore, it can be utilized for the evaluation of the free scavenging activity. The assay was performed according to the methodology previously published [67,68,69].

Briefly, the reaction mixture solution consisted of 2.7 mL of DPPH solutions (40 μg/mL) in methanol and 300 μL of HECA (10^3^ to 7.0 μg/mL) that was mixed thoroughly and incubating in darkness. After 30 min, the decline of radical concentration was measured by spectrophotometry visible at 517 nm using Biospectro SP-22. The experiment was performed in triplicate, and the mean absorption was analyzed for each concentration. Methanol was taken as the control, and the gallic acid (Sigma-Aldrich^®^, Missouri EUA, MO, USA) in the same concentrations of the extract was taken as the reference standard compound. The percentage of antioxidant activity was calculated according to Cefali et al. [67].

Scavenging percentage (%) = 1 − (*Abs*_(*treatment*)_/*Abs*_(*control*)_) × 100 (3)

where *Abs _(control_*_)_ and *Abs*_(*treatment*)_ is the absorbance of the control and the treatment respectively. To establish the half-maximal inhibitory concentration (IC_50_) of DPPH free scavenging, the samples were tested in serial dilutions (7, 15, 25, 50, 75, 100, 125, 250, 500 and 1000 μg/mL) and analyzed by linear regression models with exponential specification, the results were evaluated by one way of variance (ANOVA) followed by Tuckey test at *p* < 0.05.

### 3.3. Molecular Modeling

#### 3.3.1. Maps of Molecular Electrostatic Potential (MEP) and Frontier Orbital’s (HOMO and LUMO)

Molecular modeling started with the construction of the 12 identified compounds using GaussView 3.0 [70], and all computational calculations were performed using the Gaussian 09 program [71]. The 12 compounds identified in the leaves of *C. americana* were optimized in the density functional theory method (DFT) in theory level B3LYP/6-31G** and the frequencies were also calculated in the same method, there were no negative frequencies, thereby ensuring the minimum energy structure. After, the MEP’s generated from the atomic charge. The constructions of the MEPs and the frontier orbitals (HOMO and LUMO) were visualized with the aid of Molekel program [72].

The atomic charges used in this study were obtained with the keyword POP = CHELPG using the electrostatic potential [73]. With this strategy, it was possible to obtain the best potential quantum molecular series of points defined around the molecule, and atomic charges offer the general advantage of being physically more satisfactory than Mulliken charges [74]. The descriptors represent different sources of chemical information (features) regarding molecules that are important for the quantitative description of the molecular structure and to finding appropriate predictive models [75].

#### 3.3.2. Multivariate Analysis PCA and HCA

The analysis of inters sample and intervariable relationships were performed via Principal Component Analysis (PCA) and Hierarchical Cluster Analysis (HCA). PCA was used to reduce the number of variables and select the most relevant properties to the classification of the compounds into two groups (more stable and less stable) based in the quantum chemical analysis of the gap properties of the gallic acid compound, because the gap value indicates chemical reactivity and molecular stability [76].

After that, the structures were determined in three dimensions (3D), twelve quantum chemical and seven QSAR descriptors were selected to construct a data matrix. The QSAR descriptors included, i.e., total surface area (TSA), molecular volume (MV), molar refractivity (MR), molar polarizability (MP), coefficient of lipophilicity (LogP), molecular mass (MM) and hydration energy (HE) according to the HyperChem 6.02 [77]. The molecular descriptors were selected to provide valuable information about the influence of electronic, steric, hydrophilic and hydrophobic features, according to studies realized by Santos et al. [42].

The quantum chemical descriptors were: energy (TE), energy of the highest occupied molecular orbital (HOMO), a level below the energy of the highest occupied molecular orbital (HOMO-1), two level below the energy of the highest occupied molecular orbital (HOMO-2), lowest unoccupied molecular orbital energy (LUMO), a level above the energy of the lowest unoccupied molecular orbital (LUMO + 1), two level above the energy of the lowest unoccupied molecular orbital (LUMO + 2), difference in energy between HOMO and LUMO (GAP = HOMO − LUMO), Mulliken electronegativity (χ), molecular hardness (η), molecular softness (1/η) and Dipolo moment total (DMT).

The descriptors selected by PCA were used to perform the HCA, which was used in processing in an autoscale with the Euclidean distance metric and Incremental Linkage method. The objective of HCA was to show the compounds distributed in groups more and less stables for confirming of the PCA results. The multivariate data analysis was performed by employing the Pirouette 3.01 [78].

#### 3.3.3. Theoretical Mechanism to the Antioxidant Activity

In this work, the geometry optimization of the flavonoid derivatives has been carried out using density functional theory (DFT). The calculations were performed with the Gaussian 09 molecular package [71] and prior to any DFT calculations; all structures were submitted to PM3 [79] geometry conformational search. After the PM3 initial optimizations, the structures were reoptimized using the B3LYP/6-31G** level of theory [80]. We calculated for compounds **7** (procyanidin), **11** (galic acid) and **12** (foeniculin), the following properties: (i) ionization potential (IP) and (ii) spin density, as described by Mendes et al. [53] and Borges et al. [81]. The IP was calculated as the energy difference between a neutral molecule and the respective cation free radical as showed below:

*IP* = *EX*^●+^ − *EX* (4)

#### 3.3.4. Molecular Docking Study

Molecular docking simulation between xanthine oxidase enzyme, chemical constituents from *C. americana,* and control ligands were undertaken via AutoDock 4.2/Vina 1.1.2 by PyRx 0.8 software with default parameters by the genetic algorithm, following the protocol described by Pereira et al. and Padilha et al. [82,83]. The population size was 100, selection-pressure 1.1, the number of operations was 10,000, the number of islands was 1, the niche size was 2, operator weights for migrating was 0, mutate was 100, and crossover was 100. The coordinates *X* = 44.0000, *Y* = 34.0000 and *Z* =24.000 (grid box) from the pocket of interest was chosen based on interactions between the amino acids and a 10 Å radius sphere were defined. Ten solutions were calculated for each chemical constituent, and minimum binding energy conformations were analyzed to evaluate the best binding free energy (ΔG), binding affinity and the selectivity of the chemical constituents in therapeutic targets.

## 4. Conclusions

In the present study, the DPPH assay was used to evaluate the antioxidant activity of *Curatella americana* L., then the study of quantum chemicals was performed to obtain a relation between electronic properties and antioxidant capacity of the chemical constituents. It was possible to characterize the compounds as well as their characteristics of electro donor/electro accepter compared to the gallic acid standard. Multivariate analysis (PCA and HCA) allowed us to identify chemical descriptors that may be related to the antioxidant activity by grouping the most reactive compounds with gallic acid. The Gap and softness descriptors were shown to be important in the grouping of known antioxidant compounds such as procyanidin and quercetin and its derivatives, demonstrating that less stable molecules, that is, lower Gap values, are more antioxidant because they are more reactive. In addition, these compounds presented higher softness, especially procyanidin, indicating ease for the transition electron, confirmed by the calculations of IP and spin density. Therefore, descriptors such as Gap and softness allied to the electronic characteristics of the molecule can be used as criteria for the selection of potentially antioxidant compounds quickly and efficiently even for those compounds with recognized antioxidant activity via hydrogen abstraction as the phenolics.

The evaluation of the species regarding the possibility of prevention of the formation of ROS investigated by molecular docking simulations with XO confirmed the data from the literature showing the potential inhibitory activity of XO by the quercetin. However, we did not relate quantum chemical characteristics to this inhibitory potential.

The presence of important antioxidant compounds in the *C. americana* L. species can explain the pharmacological actions described in scientific research and in folk medicine. Therefore, it would be imperative to investigate the potential of this species and the main compounds as a natural antioxidant for the use in the pharmaceutical industry.

## Figures and Tables

**Table 1:** Descriptors most relevant for the principal component analysis.

Compounds	HE (kcal/mol)	LogP	DMT (Debye)	GAP (eV)	1/η (eV)
**1**	−40.230	−5.100	2.1057	0.462	4.3285
**2**	−31.530	−4.010	6.6104	0.447	4.4653
**3**	−42.990	−5.480	5.7241	0.194	10.2795
**4**	−39.770	−5.100	6.6674	0.469	4.2559
**5**	−42.959	−5.480	5.6174	0.568	3.5167
**6**	−37.220	−4.690	6.3514	0.186	10.6985
**7**	−49.340	−5.910	4.8393	0.064	30.8818
**8**	0.250	8.090	1.5464	6.817	0.2934
**9**	−3.510	7.260	2.3235	6.643	0.3010
**10**	−0.640	8.030	1.5438	7.071	0.2828
**11**	−25.049	−2.090	3.7109	1.474	1.3568
**12**	−1.060	2.530	1.7570	0.185	10.7611

HE: Hydration energy; LogP: lipophilicity coefficient; DMT: Dipole moment total; 1/η: molecular softness; GAP: stability measure.

**Table 2:** Theoretical Properties obtained for the compounds studied.

Molecules	HOMO (eV)	E_Neutro_ (Kcal/mol)	E_Cation_ (Kcal/mol)	IP (Kcal/mol)
**7**	−0.0435	−1340,833.36	−1340,680.69	152.67
**11**	−1.2327	−405,680.81	−405,505.18	175.62
**12**	−0.3429	−388,768.73	−388,611.82	156.90

E_Neutron_: neutro energy; E_Cation_: Cation energy.

**Table 3:** Interactions between ligands with therapeutic target XO (organism *Boss taurus*).

	Amino Acid	Distance (Å)	Type	Binding Free Energy (kcal/mol)
2 vs. XO	Leu873	3.5408	Pi-Sigma	−6.76
		4.8720	Pi-Alkyl	
	Ser876	2.3000	Conventional Hydrogen Bond	
	Phe914	3.7825	Pi-Pi Stacked	
	Thr1010	2.0916	Conventional Hydrogen Bond	
	Val1011	5.2238	Pi-Alkyl	
		5.4506	Pi-Alkyl	
	Leu1014	5.0207	Pi-Alkyl	
		2.8423	Pi-Sigma	
11 vs. XO	Phe914	3.6159	Pi-Pi Stacked	−4.4
	Phe1009	5.4325	Pi-Pi T-shaped	
	Thr1010	2.0504	Conventional Hydrogen Bond	
		2.8382	Conventional Hydrogen Bond	
	Ala1078	4.9487	Pi-Alkyl	
	Ala1079	3.6445	Pi-Sigma	
	Glu1261	1.9196	Conventional Hydrogen Bond	
	Leu873	4.4308	Alkyl	
		4.9122	Alkyl	
	Phe914	3.5349	Pi-Pi Stacked	
12 vs. XO	Phe1009	4.8397	Pi-Pi Stacked	−7.13
	Val1011	4.4798	Alkyl	
	Ala1078	4.9457	Pi-Alkyl	
		4.1350	Alkyl	
	Ala1079	4.1423	Pi-Alkyl	
HPX vs. XO	Glu802	3.2579	Conventional Hydrogen Bond	−5.65
	Arg880	3.0805	Conventional Hydrogen Bond	
	Phe914	3.4234	Pi-Pi Stacked	
		3.8092	Pi-Pi Stacked	
	Phe1009	4.7907	Pi-Pi T-shaped	
		5.2023	Pi-Pi T-shaped	
	Thr1010	3.1183	Conventional Hydrogen Bond	
		2.8251	Conventional Hydrogen Bond	
	Ala1078	4.6549	Pi-Alkyl	
	Ala1079	3.9407	Pi-Sigma	
		4.8965	Pi-Alkyl	
FBX vs. XO	Glu802	1.9544	Conventional Hydrogen Bond	−6.1
	Leu873	3.7514	Pi-Sigma	
	Ser876	2.8449	Conventional Hydrogen Bond	
	Phe914	3.8848	Pi-Pi Stacked	
	Phe1005	3.8139	Pi-Alkyl	
		4.6647	Pi-Alkyl	
	Phe1009	4.4818	Pi-Pi T-shaped	
		5.5513	Pi-Sulfur	
	Val1011	4.8667	Pi-Alkyl	
	Leu1014	4.2479	Pi-Alkyl	
	Ala1078	4.4684	Pi-Alkyl	
	Ala1079	4.7224	Pi-Alkyl	
		3.7013	Alkyl	

## Supplementary Materials

The following are available online at http://www.mdpi.com/1424-8247/11/3/72/s1. Figure S1: Potential electrostaic maps of the identified compounds in the *Curatella amerina* L*.* leaves; Figure S2: The transition status of frontier orbital (HOMO and LUMO) compounds **2**, **7**–**12**.
